# Supplementary material for: Breaking through the Mermin-Wagner limit in 2D van der Waals magnets
Source: Nat Commun. 2022 Nov 14;13:6917. doi: 10.1038/s41467-022-34389-0 (PMC9663506; doi:10.1038/s41467-022-34389-0)
Supplement: Supplementary file 1 — Supplementary Information [file 41467_2022_34389_MOESM1_ESM.pdf]

Supplementary Information:
Breaking through the Mermin-Wagner limit in 2D van der Waals magnets

Sarah Jenkins[1,2,3], Levente Rózsa[4], Unai Atxitia[5,6], Richard F. L. Evans[1], Kostya S. Novoselov[7] Elton J. G. Santos[8,9,10†]

[1] *Department of Physics, University of York, York, YO10 5DD, UK*

[2] *TWIST Group, Institut für Physik, Johannes Gutenberg Universität, 55128 Mainz, Germany*

[3] *TWIST Group, Institut für Physik, Universität Duisburg-Essen, Campus Duisburg, 47057 Duisburg, Germany*

[4] *Fachbereich Physik, Universität Konstanz, D-78457 Konstanz, Germany*

[5] *Dahlem Center for Complex Quantum Systems and Fachbereich Physik, Freie Universität Berlin, 14195 Berlin, Germany*

[6] *Instituto de Ciencia de Materiales de Madrid, CSIC, Cantoblanco, 28049 Madrid, Spain*

[7] *Institute for Functional Intelligent Materials, National University of Singapore, 117544, Singapore*

[8] *Institute for Condensed Matter Physics and Complex Systems, School of Physics and Astronomy, The University of Edinburgh, EH9 3FD, United Kingdom*

[9] *Donostia International Physics Center (DIPC), 20018 Donostia-San Sebastián, Basque Country, Spain*

[10] *Higgs Centre for Theoretical Physics, The University of Edinburgh, EH9 3FD, United Kingdom*

[†] *Corresponding author: esantos@ed.ac.uk*

Supplementary Section 1: Large-scale atomistic simulations

The system is integrated using a Monte Carlo integrator to find the ground state. The Metropolis algorithm [1] takes a random spin with direction $\mathbf{S}_i$, and changes it to $\mathbf{S}'_i$ - the trial spin direction. The next step is to calculate the change in energy between the initial and trial states $\Delta E = E(\mathbf{S'}_i) - E(\mathbf{S}_i)$. The new trial direction is then either accepted or rejected based on a probability (P):

$$P = \exp\left(-\frac{\Delta E}{k_B T}\right), \tag{1}$$

where k_B is the Boltzmann constant and T is the absolute temperature. If the change in energy is less than zero then the spin is automatically accepted. This is repeated N times per Monte Carlo step where N is the number of atoms in the system.

The trial spin positions are selected using a sampling method[2] with three types of trial move: spin flip, Gaussian and random. The three types of trial moves allow the system to equilibrate quickly at any temperature. At high temperatures the random and spin flip sampling steps allow large changes in the spin direction and at low temperatures the Gaussian sampling creates small changes in the spin direction. The data files generated in this research can be found at the following repository link.

Supplementary Section 2: Time-intensive computational simulations

The simulations were run on the EPSRC Tier-2 National HPC CIRRUS (project ec131) and the UK National Supercomputer ARCHER2 (project d429). The largest 1000 nm × 1000 nm simulations

comprised over 8 million atoms. To fully equilibrate the systems, large number of Monte Carlo steps are required. We have used atomistic methods[3,4] with large parallelisation features to over 6000 CPU cores. For a single temperature point on the magnetisation vs temperature curves a simulation of 40 million Monte Carlo steps was performed. For the largest systems, 432 CPU cores of CIRRUS/ARCHER2 were used and converged on an average of 30 hours. This is nearly 113,000 hours of simulation time per point on the plots and each snapshot in Fig. 4 in the main text.

Supplementary Section 3: Size effects and anisotropy

The intrinsic magnetisation was simulated as a function of temperature with ($K = 1 \times 10^{-24}$ J/atom) and without ($K = 0$) anisotropy for a flake size of $100\times$ 100 nm^2 (Supplementary Figure 1). The data show that there is a smaller change in the crossover temperature than for the $1000\times$ 1000 nm^2 flake size (Fig. 1**b**) when fitted by Eq. (3) in the main text. The lack of anisotropy decreases the exponent: without anisotropy the fitting parameters are $\beta = 0.53 \pm 0.03$ and $T_x = 26.64 \pm 0.31$ K and with anisotropy $\beta = 0.47 \pm 0.02$ and $T_x = 27.14 \pm 0.27$ K.

Supplementary Section 4: The choice of Monte Carlo trial moves and dipolar interactions

We have used a Monte Carlo algorithm which chooses the new trial positions uniformly over the three possible trial moves[5]. Here we compare the results to an adaptive algorithm[6], which adapts the choice of trial move to the simulation to improve the acceptance probability. Both simulations show the same magnetisation vs. temperature profile with the same crossover temperature (Sup-

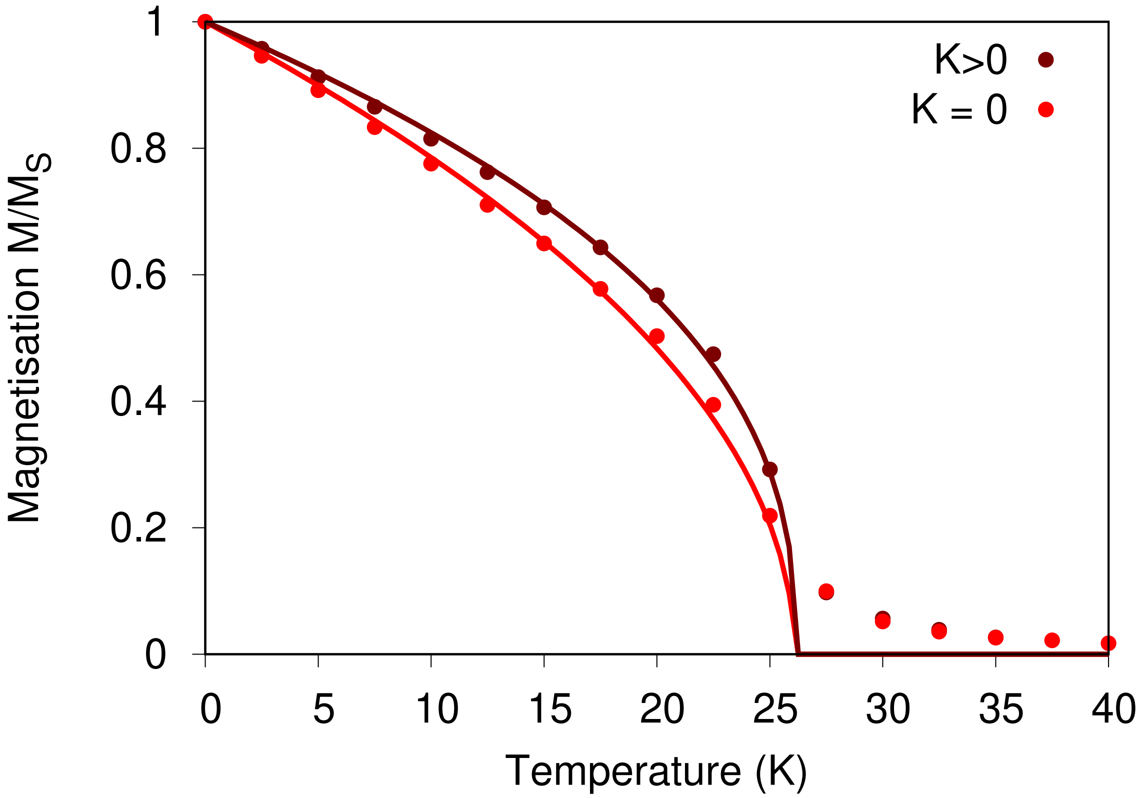

Supplementary Figure 1: **The effect of anisotropy for a flake size of 100 $\times$ 100 nm^2.** See the caption of Figure 1**b** for the simulation parameters.

plementary Fig. 2**a-b**). However, the adaptive algorithm converges approximately 10 times faster. In the simulations in the paper we have started the system in a fully ordered state. In Supplementary Fig. 2**c** we show that the magnetisation vs. temperature curves are the same for both the fully ordered and the fully disordered initial states and in Supplementary Fig. 2**d** we show the ordering process at $T = 10$ K starting from random and ordered magnetic states. The ordering process from the disordered state is slow due to the large size of the system, taking around 30×10^6 Monte Carlo steps to reach thermal equilibrium, but exhibits a stable average magnetisation length for the last 10×10^6 Monte Carlo steps. For the final 10^6 steps, the average magnetisation is the same as that

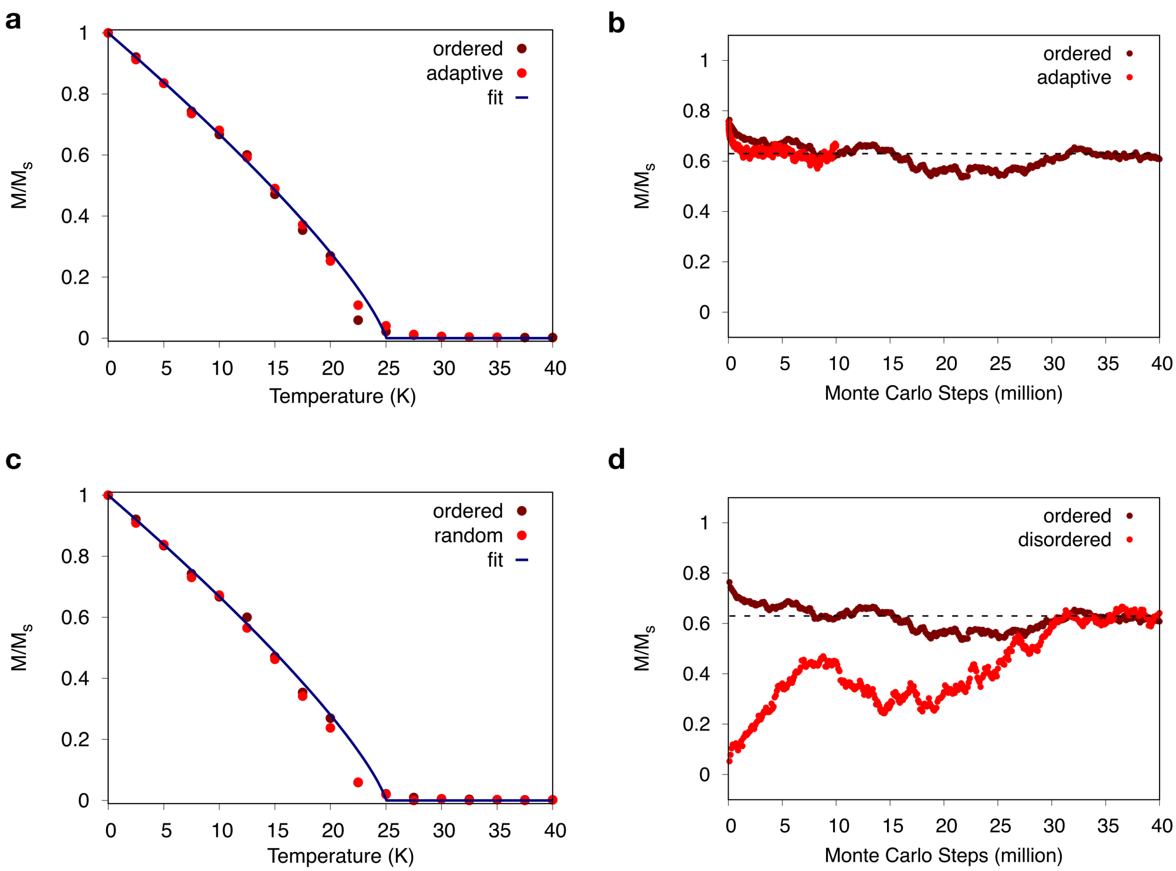

Supplementary Figure 2: **Comparison of different simulation methods. a,** Simulated temperature-dependent intrinsic magnetisation using the uniform choice between the trial steps and the adaptive algorithm, respectively. The data show that the equilibrium intrinsic magnetisation at each temperature is the same independent of the algorithm, with the same crossover temperature around $T_x \sim 23$ K when fitted by Eq. (3) with fitting parameter $\beta = 0.71$. **b,** The intrinsic magnetisation at $T = 10$ K over 40×10^6 Monte Carlo steps using the uniform choice between the trial steps and the adaptive algorithm, respectively. Both situations magnetise to approximately $|m|$ =0.64, with the adaptive algorithm converging slightly faster. **c,** Comparison between fully ordered and fully random starting configurations for the intrinsic magnetisation. **d,** Convergence of the intrinsic magnetisation to equilibrium for the fully ordered and fully random starting configurations.

obtained from the fully ordered starting state.

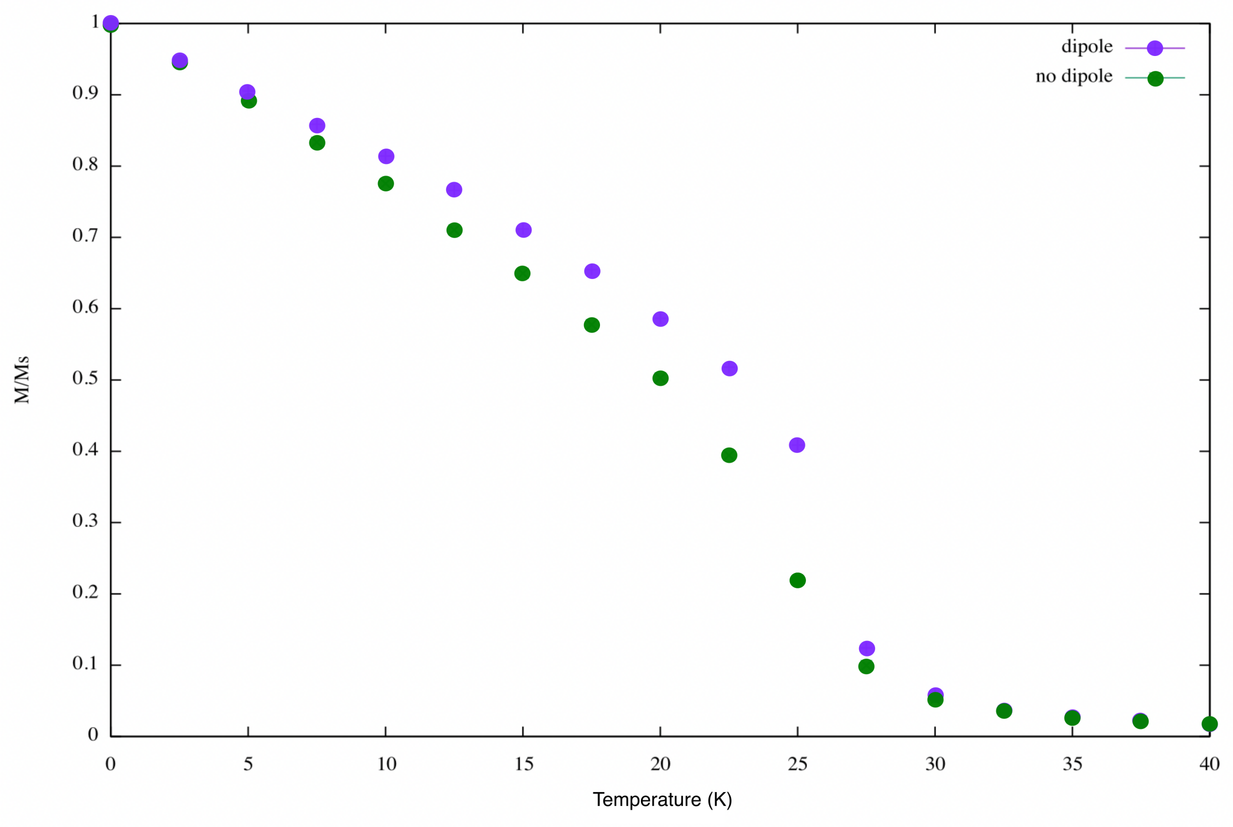

Supplementary Figure 3: **Influence of the dipolar interactions.** Variation of the intrinsic magnetisation M/M_S for a 2D honeycomb lattice of 50 nm with and without dipole-dipole interactions.

Supplementary Section 5: Angular distribution of the spins

The final spin configurations for the 1000×1000 nm^2 flake after 40 million Monte Carlo steps for each size and temperature are shown in Fig. 1. In Supplementary Fig. 4, we plot the distribution of the angle from the average magnetisation direction of the system over the lattice sites. A deviation from the sin-like behaviour indicates the presence of short-range order in the system.

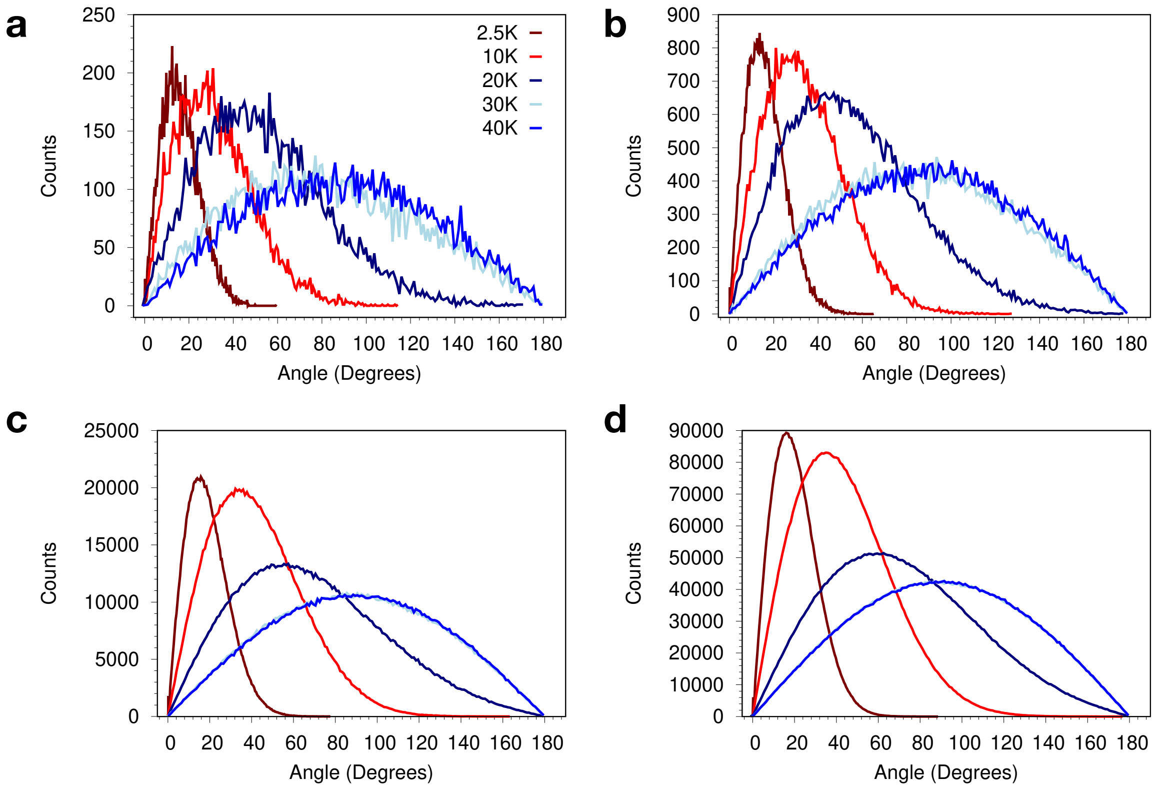

Supplementary Figure 4: **The distribution of the angle measured from the mean magnetisation direction of the system.** **a,** For $50{\times}50$ nm^2, **b,** $100{\times}100$ nm^2, **c,** $500{\times}500$ nm^2, and **d,** $1000{\times}1000$ nm^2 flake sizes. As there is no anisotropy, the average direction is random and can assume any position in space. The small width of the distribution shows that short-range order persists up to 20 K even up to a system size of 1000×1000 nm^2.

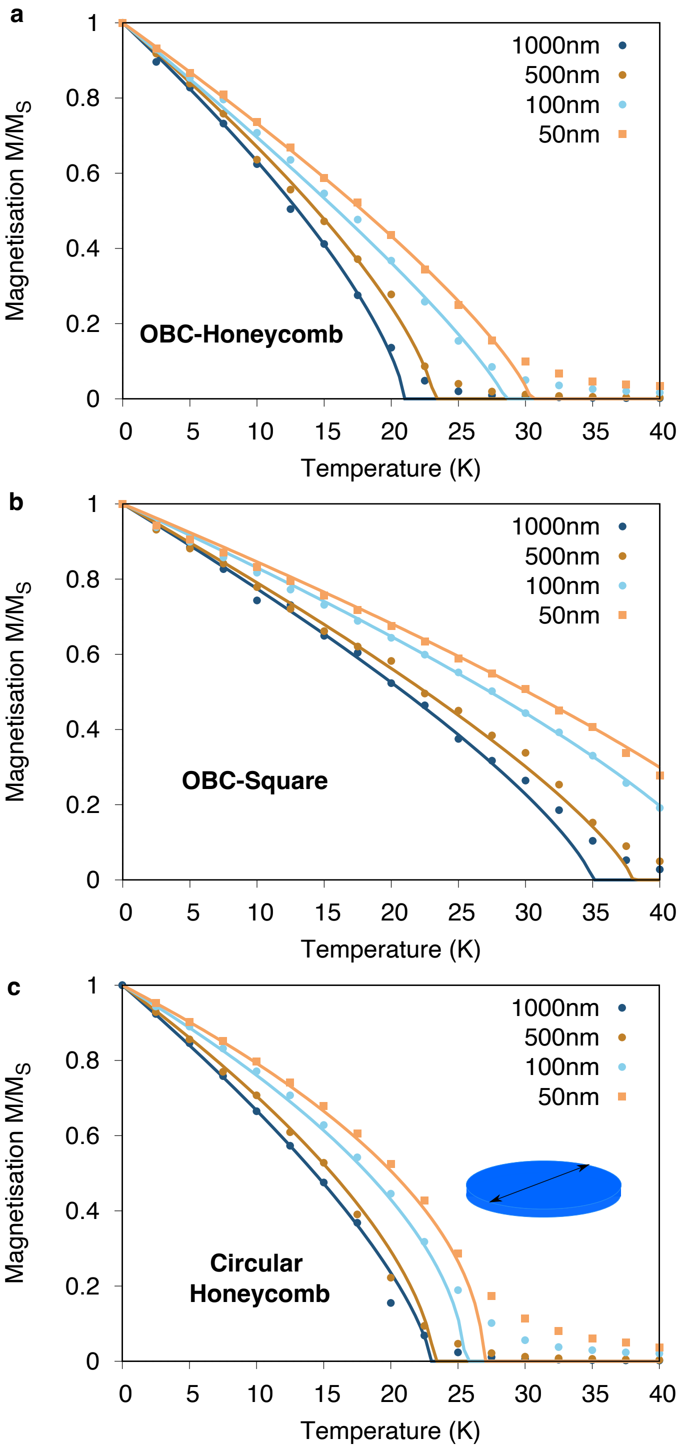

Supplementary Figure 5: **Different boundary conditions and circular flake. a-c,** Open boundary conditions (OBC) applied to honeycomb and square lattices, respectively. Similar approaches and theoretical setup is used for these simulations as in Figure 2. **e,** Finite circular flake with honeycomb lattice at different diameters showed in the inset. Points are simulated data and solid lines fitting curves to Eq. 3. We noticed the neither the OBCs nor the flake shape change the conclusions.

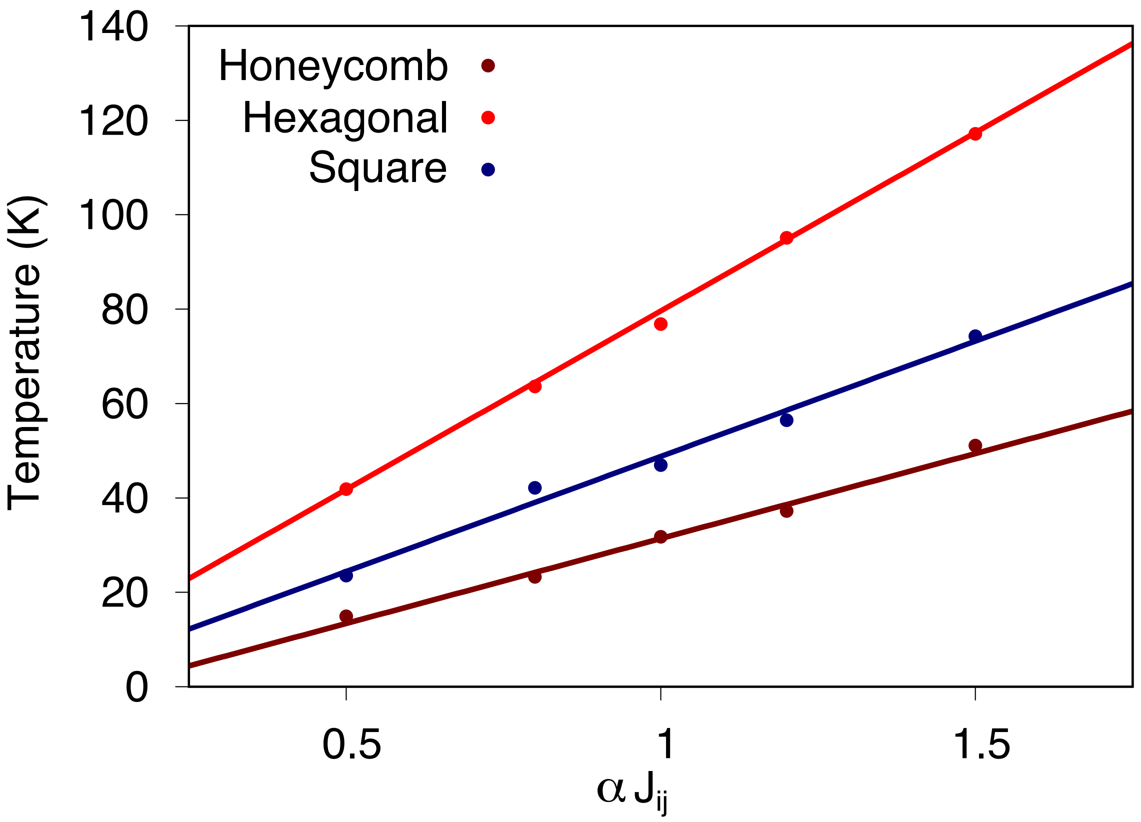

Supplementary Figure 6: **Strength of the exchange interactions.** Calculated crossover temperature (K) versus the exchange interactions J_{ij} multiplied by the numerical factor α shown in the x-axis. α either increased or decreased the magnitudes of J_{ij} relative to their pristine values (e.g. $1 \times J_{ij}$) taken into account in the computations. A linear variation is observed between the crossover temperatures and αJ_{ij} which indicates that the stabilisation of 2D magnetism is independent of the strength of exchange interactions considered. Similar approaches as those used in Figure 2 are considered with a flake of 100 nm.

Supplementary Section 6: Anisotropic spherical model

We use the anisotropic spherical model (ASM) for the calculation of the finite-size and boundary effects on the intrinsic magnetization[7,8]. Previous works have demonstrated that differently to spin-wave theory (SWT), the ASM takes into account Goldstone modes in the system and self-consistently generates a gap in the correlation functions which avoids the infrared divergences. The Goldstone modes prevent phase transitions for isotropic systems in dimensions $d \leq 2$. The ASM has been used mostly for the analytical study of magnetisation in thin films[9] and domain walls[7]. So far, comparison of the theory to computational results is restricted to small 3D magnets of around a few nanometers lateral size in simple cubic lattices[8], where good agreement has been found. Here, we adapt the work by Kachkachi and Garanin in Ref. [8] to 1D and 2D systems for the isotropic Heisenberg Hamiltonian in the absence of an applied magnetic field. We consider a 1D spin chain, and three different lattices for the 2D systems; cubic, hexagonal and honeycomb.

The average magnetisation is defined as

$$\mathbf{m} = \frac{1}{N}\sum \mathbf{m}_i, \tag{2}$$

which vanishes for finite-size systems in the absence of magnetic field due to the Goldstone mode corresponding to the global rotation of the magnetisation. However, at reduced temperatures the spins in the system are aligned with respect to each other. Thus, a finite intrinsic magnetisation M exists,

$$M = \sqrt{\langle \left(\frac{1}{N}\sum_i \mathbf{S}_i \right)^2 \rangle}, \tag{3}$$

corresponding to the quantity calculated using Monte Carlo simulations in the main text.

The Hamiltonian is

$$\mathscr{H} = -\sum_{i<j} J_{ij}\mathbf{S}_i \cdot \mathbf{S}_i. \tag{4}$$

For the sake of simplicity, we reduce the energy scale to dimensionless variables, $\lambda_{ij} = J_{ij}/\sum_j J_{ij}$, and the reduced temperature is defined as $\theta = T/T_c^{\mathrm{MFA}}$. For direct comparison to simulations using classical spin models, one needs to renormalize T_c^{MFA} adequately. In this work we find that its value needs to be reduced compared to the analytical expression $T_c^{\mathrm{MFA}} = \sum_j J_{ij}/3$ to achieve good quantitative agreement with the simulations. In Fig. 2(a)-(c) of the main text, the shaded areas denote a range where the rescaling factor is varied between 0.65 and 0.85. We attribute the necessity of the rescaling to the fact that the ASM becomes exact as the spin dimension D becomes infinite, while the simulations are carried out for $D = 3$.

The anisotropic spherical model consists of self-consistency equations for the magnetization components, which are $m_i^\alpha = \langle S_i^\alpha \rangle = 0$ ($\alpha = x, y, z$) vanishing in the absence of an applied field, and the Dyson equation for the correlation function $s_{il} = D\langle S_i^\alpha S_l^\alpha \rangle$:

$$s_{il} = \theta G_i \delta_{il} + G_i \sum_j \lambda_{ij} s_{jl} \tag{5}$$

where δ_{il} is the Kronecker symbol. G_i is the so-called gap parameter to be determined from the set of constraint equations $s_{ii} + \mathbf{m}_i^2 = 1$ on all sites $i = 1, \ldots, N$ of the lattice. This reduces to $s_{ii} = 1$ for zero external field. Solving these equations consists of determining the correlation function s_{ij} as a function of G_i from Eq. (5) and using $s_{ii} = 1$ in order to obtain G_i. Therefore, the number of parameters in the calculations is $N^2 + N$, which can be reduced by taking symmetries into account. For periodic boundary conditions (pbc) with translational symmetry, only the sublattices have to be

treated separately. This allows for an analytical treatment and to study finite-size effects separately from boundary effects. In Fourier space, Eq. (5) simplifies to

$$s_{\mathbf{k},qq'} = \theta \left(\frac{1}{G_q} \delta_{qq'} - \lambda_{\mathbf{k},qq'} \right)^{-1}, \tag{6}$$

where q and q' denote sublattice indices. The case of a single sublattice has been treated in Ref. [8], which is valid for the chain, the square and the hexagonal lattice studied here. However, for the honeycomb lattice two sublattices have to be taken into account. The constraint equations are given by

$$s_{ii} = \frac{1}{\mathscr{N}_c} \sum_{\mathbf{k}} \theta \left(\frac{1}{G_q} \delta_{qq'} - \lambda_{\mathbf{k},qq'} \right)^{-1} \Bigg|_{q=q'} = 1, \tag{7}$$

where $\mathscr{N}_c$ is the number of cells, over which the Fourier transformation is performed.

For a single sublattice, one can then calculate the intrinsic magnetization M:

$$M = \sqrt{1 - \theta G \tilde{P}_N(G)}, \tag{8}$$

with

$$\tilde{P}_N(G) \equiv \frac{1}{N} {\sum_{\mathbf{k}}}' \frac{1}{1 - G\lambda_{\mathbf{k}}} \tag{9}$$

where the prime indicates that the mode $\mathbf{k} = 0$ is excluded from the sum. At low temperatures in zero field M deviates from 1 according to the law

$$M \cong 1 - \theta W_N^{\text{pbc}}/2 \tag{10}$$

where the coefficient of the term linear in θ is smaller than in the bulk. The coefficient W_N^{pbc} reads

$$W_N^{\text{pbc}} \equiv \frac{1}{N} {\sum_{\mathbf{k}}}' \frac{1}{1 - \lambda_{\mathbf{k}}}. \tag{11}$$

In particular, in two dimensions one has $W(\text{pbc}) \cong 8\pi \ln L + \text{const}$, which results in

$$M \cong 1 - \theta(4\pi \ln L + \text{const}). \tag{12}$$

In the following, the geometrical factors $\lambda_{\mathbf{k}}$ are provided for different lattice structures.

Chain: For a one-dimensional system along the x axis with $N = L$ spins, the wave vector is defined as

$$k_x = \frac{2\pi n_x}{N}, \quad n_x = 0, \ldots, N-1. \tag{13}$$

The reduced coupling is $\lambda_{\mathbf{k}} = \cos(k_x)$.

In Fig. 4(d) of the main text, the results of the ASM with periodic boundary conditions are compared to the analytical solution of the nearest-neighbour classical Heisenberg model with free boundary conditions[10]. Introducing

$$L(X) = \frac{1}{\tanh(1/X)} - X, \tag{14}$$

where $X = \frac{2}{3}\theta$, the intrinsic magnetization is then calculated as

$$M = \frac{1}{N}\sqrt{\frac{N(1+L)}{1-L} - \frac{2L(1-L^N)}{(1-L)^2}}. \tag{15}$$

Square lattice: The wave vectors are defined as

$$k_\alpha = \frac{2\pi n_\alpha}{L_\alpha}, \quad n_\alpha = 0, \ldots, L_\alpha - 1, \alpha = x, y. \tag{16}$$

The reduced couplings $\lambda_{\mathbf{k}} = \frac{1}{2}(\cos(k_x) + \cos(k_y))$ are used in Eq. (6).

Hexagonal lattice: The wave vectors are defined as

$$k_x = \frac{2\pi n_x}{L_x}, \qquad n_x = 0, \ldots, L_x - 1, \tag{17}$$

$$k_y = -\frac{\sqrt{3}}{3}\frac{2\pi n_x}{L_x} + \frac{2\sqrt{3}}{3}\frac{2\pi n_y}{L_y}, \qquad n_\alpha = 0, \ldots, L_\alpha - 1, \qquad \alpha = x, y. \tag{18}$$

The reduced couplings $\lambda_{\mathbf{k}}$

$$\lambda_{\mathbf{k}} = \frac{1}{2}\left[\cos(k_x) + 2\cos(\frac{1}{2}k_x)\cos(\frac{\sqrt{3}}{2}k_y)\right] \tag{19}$$

are used in Eq. (6).

Honeycomb lattice: The wave vectors are defined as

$$k_x = \frac{1}{3}\frac{2\pi n_x}{L_x} + \frac{1}{3}\frac{2\pi n_y}{L_y}, \qquad n_\alpha = 0, \ldots, L_\alpha - 1, \qquad \alpha = x, y; \tag{20}$$

$$k_y = \frac{\sqrt{3}}{3}\frac{2\pi n_x}{L_x} - \frac{\sqrt{3}}{3}\frac{2\pi n_y}{L_y}, \qquad n_\alpha = 0, \ldots, L_\alpha - 1, \qquad \alpha = x, y. \tag{21}$$

The reduced couplings $\lambda_{\mathbf{k}}$ read

$$|\lambda_{\mathbf{k}}| = \frac{1}{3}\sqrt{(\cos(k_x) + 2\cos(\frac{1}{2}k_x)\cos(\frac{\sqrt{3}}{2}k_y))^2 + (\sin(k_x) - 2\sin(\frac{1}{2}k_x)\sin(\frac{\sqrt{3}}{2}k_y))^2}, \tag{22}$$

and are only finite between the two sublattices. In Eq. (7) one can use

$$\left.\left(\frac{1}{G_q}\delta_{qq'} - \lambda_{\mathbf{k},qq'}\right)^{-1}\right|_{q=q'} = \frac{G}{1 - G^2\,|\lambda_{\mathbf{k}}|^2}, \tag{23}$$

where G is the same for both sublattices. The intrinsic magnetisation of the honeycomb lattice is calculated as

$$M = \sqrt{\frac{\theta G}{N(1-G)}}, \tag{24}$$

where N is the total number of sites rather than the number of cells. This can be derived from Eq. (3) using the expressions for the correlation function and $\lambda_{\mathbf{k}=0} = 1$.

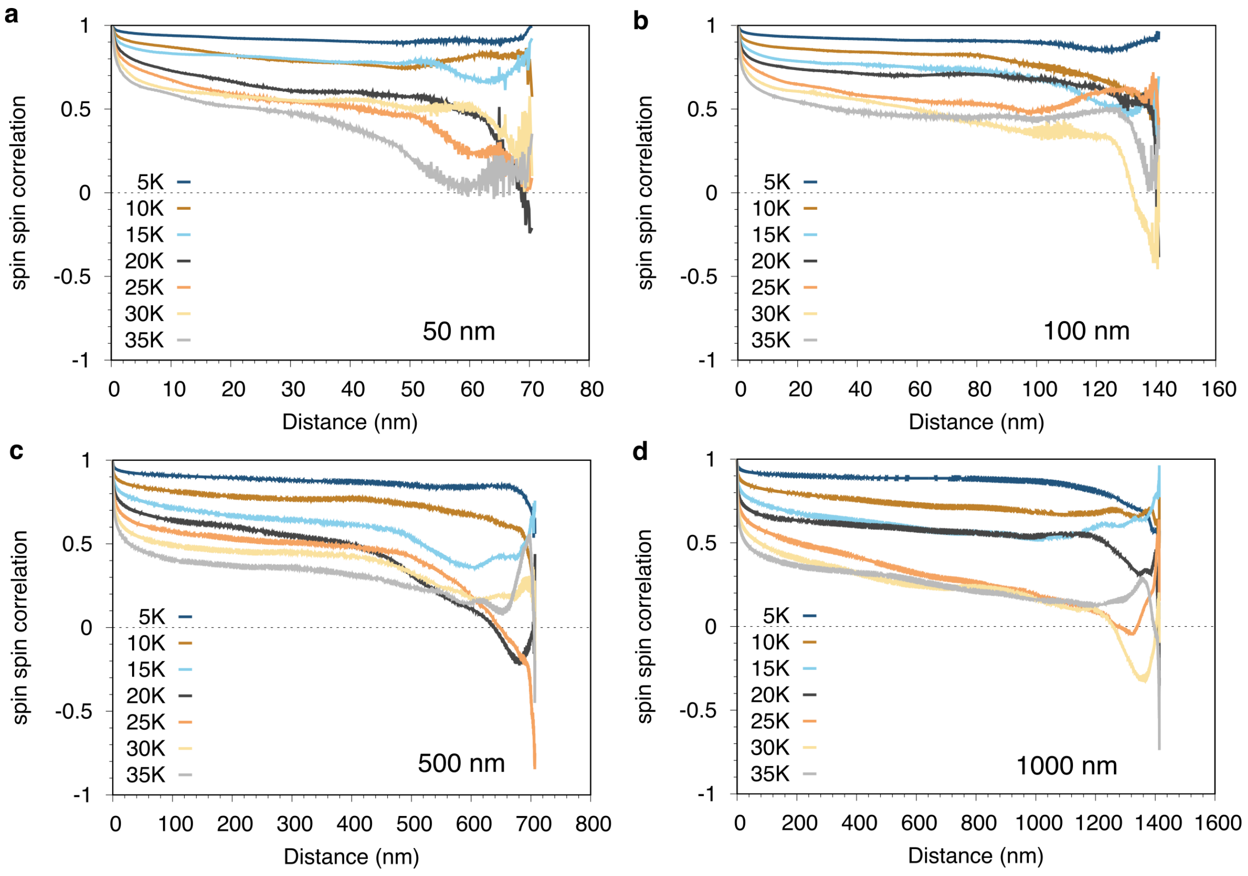

Supplementary Figure 7: **Spin-spin correlation function for a 2D hexagonal lattice.** Spin-spin correlation functions at different temperatures **a,** For 50×50 nm^2, **b,** 100×100 nm^2, **c,** 500×500 nm^2, and **d,** 1000×1000 nm^2 flake sizes, respectively. Exchange parameters are the same as used in Figure 2.

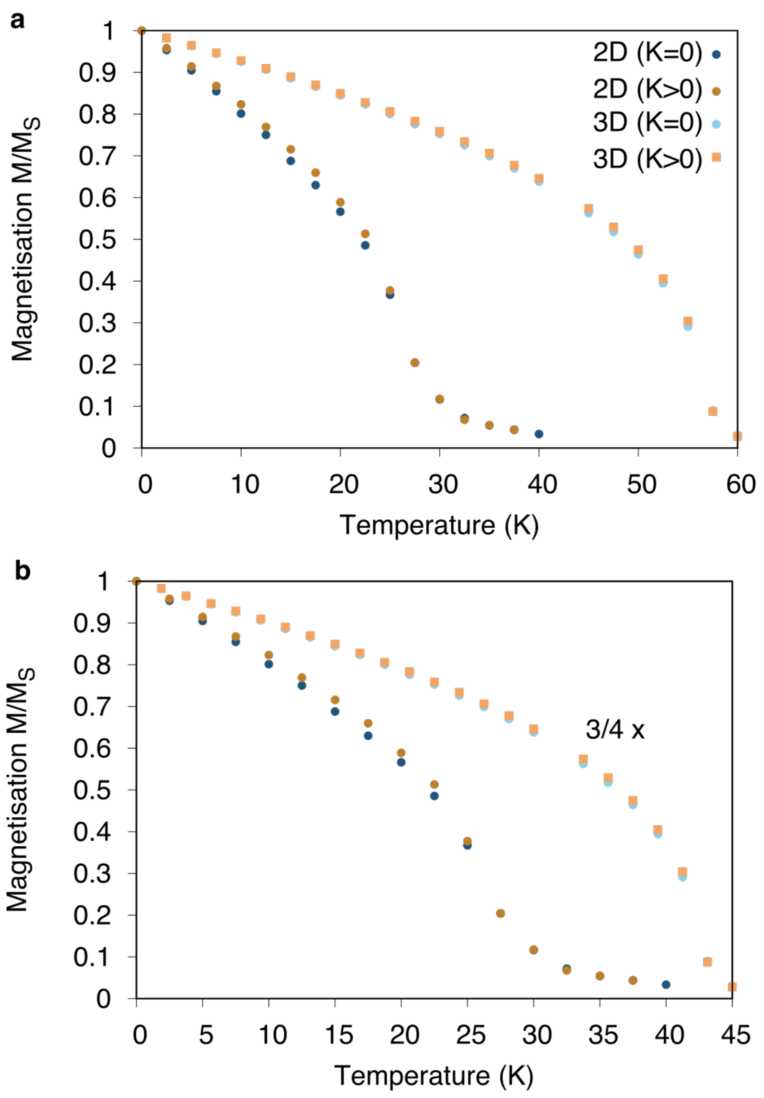

Supplementary Figure 8: **Comparison between 3D and 2D magnetisation for finite systems.** **a,** M/M$_S$ versus temperature for 2D (50 nm $\times$ 50 nm) and 3D (50 nm $\times$ 50 nm $\times$ 20 nm) finite honeycomb systems with ($K = 1 \times 10^{-24}$ J/atom) and without magnetic anisotropy ($K = 0$). **b,** Similar as **a,** but with the temperature in the 3D case scaled by a 3/4 factor to remove the effect of an additional interlayer exchange interaction taken into account. That is, if the dimensionality would be a scaling factor on the crossover temperature, both M(T) curves for 2D and 3D systems would be close or superposed to each other. However, we found that the finite volume liaised with the exchange interactions play together to stabilise higher crossover temperatures and a different shape of the M(T) between 2D and 3D systems. Indeed, we cannot separate contributions from exchange interactions and volume in a simply way which indicates that strictly 2D magnetic order is different to that at bulk compounds. Similar methods as those for Figure 1 are used.

Supplementary References

1. Metropolis, N., Rosenbluth, A. W., Rosenbluth, M. N., Teller, A. H. & Teller, E. Equation of state calculations by fast computing machines. *Journal of Chemical Physics* **21**, 1087–1092 (1953).

2. Nowak, U. & Hinzke, D. Magnetization switching in small ferromagnetic particles: Nucleation and coherent rotation. *Journal of Applied Physics* **85**, 4337–4339 (1999). URL `https://doi.org/10.1063/1.370360`. `https://doi.org/10.1063/1.370360`.

3. Evans, R. F. *et al.* Atomistic spin model simulations of magnetic nanomaterials. *Journal of Physics Condensed Matter* **26** (2014).

4. Kartsev, A., Augustin, M., Evans, R. F. L., Novoselov, K. S. & Santos, E. J. G. Biquadratic exchange interactions in two-dimensional magnets. *npj Comput. Mater.* **6**, 150 (2020). URL `https://doi.org/10.1038/s41524-020-00416-1`.

5. Nowak, U., Chantrell, R. W. & Kennedy, E. C. Monte Carlo simulation with time step quantification in terms of Langevin dynamics. *Phys. Rev. Lett.* **84**, 163–166 (2000). URL `https://link.aps.org/doi/10.1103/PhysRevLett.84.163`.

6. Alzate-Cardona, J. D., Sabogal-Suárez, D., Evans, R. F. L. & Restrepo-Parra, E. Optimal phase space sampling for Monte Carlo simulations of Heisenberg spin systems. *Journal of Physics: Condensed Matter* **31**, 095802 (2019). URL `https://doi.org/10.1088%2F1361-648x%2Faaf852`.

7. Garanin, D. A. Spherical model for anisotropic ferromagnetic films. *Journal of Physics A: Mathematical and General* **29**, L257–L262 (1996). URL http://dx.doi.org/10.1088/0305-4470/29/10/006.

8. Kachkachi, H. & Garanin, D. A. Boundary and finite-size effects in small magnetic systems. *Physica A: Statistical Mechanics and its Applications* **300**, 487–504 (2001). URL https://www.sciencedirect.com/science/article/pii/S0378437101003612.

9. Garanin, D. A. Ordering in magnetic films with surface anisotropy. *Journal of Physics A: Mathematical and General* **32**, 4323–4342 (1999). URL http://dx.doi.org/10.1088/0305-4470/32/24/301.

10. Fisher, M. E. Magnetism in one-dimensional systemsthe heisenberg model for infinite spin. *American Journal of Physics* **32**, 343–346 (1964). URL https://doi.org/10.1119/1.1970340. https://doi.org/10.1119/1.1970340.
